# Gender-Related Factors in Medication Adherence for Metabolic and Cardiovascular Health

**DOI:** 10.3390/metabo13101087

**Published:** 2023-10-17

**Authors:** Vittorio Venditti, Enrico Bleve, Susanna Morano, Tiziana Filardi

**Affiliations:** 1 Department of Experimental Medicine, “Sapienza” University of Rome, Viale Regina Elena 324, 00161 Rome, Italy; vittorio.venditti@uniroma1.it (V.V.); enrico.bleve@uniroma1.it (E.B.); susanna.morano@uniroma1.it (S.M.); 2 Department of Human Sciences and Promotion of the Quality of Life, San Raffaele Roma Open University, Via di Val Cannuta, 247, 00166 Rome, Italy

**Keywords:** gender, sex, metabolic syndrome, diabetes, hypercholesterolaemia, arterial hypertension, cardiovascular diseases, heart failure

## Abstract

This review explores the impact of gender on medication adherence in the context of metabolic and cardiovascular diseases. Optimal adherence to medication is crucial for achieving treatment goals and preventing adverse outcomes in chronic diseases. The review examines specific conditions such as type 2 diabetes, hypercholesterolemia, arterial hypertension, cardiovascular diseases, and heart failure. In type 2 diabetes, female sex, younger age, new drug prescription, non-white ethnicity, low education level, and low income were identified as predictors of non-adherence. Depressive disorders were also found to influence adherence. In hypercholesterolemia, women exhibited poorer adherence to statin therapy compared to men, with statin-related side effects and patient perception being significant factors. Adherence to anti-hypertensive therapy showed conflicting results, with studies reporting both higher and lower adherence in women. Limited evidence suggests that women may have poorer adherence after acute myocardial infarction and stroke. Regarding heart failure, adherence studies have shown inconsistent findings. The reasons for gender differences in medication adherence are multifactorial and include sociodemographic, disease-related, treatment-related, and psychological factors. This review emphasizes the need for further research to better understand these differences and develop gender-customized interventions that can improve medication adherence and reduce the burden of metabolic and cardiovascular diseases.

## 1. Introduction

The European Commission defines medication adherence as “the process by which patients take their medications as prescribed”. This process consists of three main phases: initiation, which is the period between prescription and the first dose administration; implementation, which measures the extent of adherence to the prescribed dose; and discontinuation, which refers to the cessation of therapy [1]. Obstacles to adherence can occur at any of these three phases. In fact, a significant number of patients never start a new therapy after it is prescribed [2]. On the other hand, implementation can be affected by both involuntary behaviors, such as negligence and inattention due to cognitive impairment, and voluntary actions to alter the timing and dosage of prescriptions. Although many people use the terms adherence and persistence interchangeably, they have different connotations. Persistence refers to the time between initiation and the last dose before discontinuation, and non-persistence is considered the most common cause of reduced adherence [1].

Good adherence is essential for preventing adverse outcomes and achieving therapeutic targets in chronic diseases. Therefore, accurately estimating the degree of adherence is fundamental. However, assessing patients’ adherence can be challenging for healthcare providers due to the widespread problems faced by healthcare systems worldwide, including resource and time constraints.

Various methods, both direct and indirect, can be used to determine adherence, but they are rarely employed in clinical practice due to their high costs, complexity, and lack of accuracy. Indirect methods, such as interviews, questionnaires, pill counts, and prescription refill data, are undoubtedly cheaper, less complex, and more accessible. On the other hand, direct methods, which are more reliable but complex and expensive, include drug administration supervised by physicians, electronic tools that record pill removal from packaging, digital sensors that track pill ingestion, and measurement of drug metabolites in body fluids [3].

Identifying risk factors for poor adherence plays a crucial role in the management of chronic diseases. The most significant risk factors can be categorized into four main areas: demographic factors (gender, age, education, social background), healthcare system issues or patient-physician relationships, treatment issues (therapy complexity, side effects), comorbidity (polypharmacy and other diseases), and subjective factors (consciousness, awareness of therapy benefits) [4].

It is now commonly understood, and relatively recent knowledge, that gender may influence the medication adherence process. The growing interest in sex-related approaches may pave the way for improving adherence and the management of chronic diseases through personalized treatments.

Furthermore, it is important to properly define the distinct meanings of “sex” and “gender”, which are often used interchangeably. Sex refers to the biological component regulated by sex hormones, while gender is a more complex characteristic that results from interactions between individuals and their surrounding environment [5,6]. Both sex and gender have implications for patients’ attitudes and subsequently impact disease outcomes. They are involved in various aspects of diseases, including epidemiology, pathophysiology, therapy, and outcomes.

Today, a completely new clinical approach to metabolic and cardiovascular diseases (CVD) that takes into account gender disparities is required.

The aim of this review is to analyze and present all available evidence regarding sex and gender differences in adherence to therapies for metabolic and cardiovascular diseases. Special attention will be given to emerging explanations for gender disparities. This review aims to provide clinicians with a better understanding of the clinical course of diseases and, in turn, improve the current clinical approach through gender-driven personalized treatments.

## 2. Medication Adherence in Metabolic and Cardiovascular Diseases

### 2.1. Type 2 Diabetes

According to the recent pandemic proportions of diabetes in the last decades, it is reported that almost 500 million people around the globe are affected by this chronic disease. In particular, type 2 diabetes (T2D) is estimated to cause the majority of all cases, approximately 90% [7]. Given these numbers, it can be easily understood why currently there is an increase in diabetes prevalence and mortality, with more than 4 million deaths claimed to be caused by diabetes. Several epidemiological studies have reported few sex and gender differences in T2D prevalence. In particular, a higher T2D rate has been observed in adult men compared to women of the same age [8,9], although there is an opposite trend concerning the diabetes-related mortality rate [7].

It is well established that patients with T2D have an increased risk for CVD compared to non-diabetic patients. However, the burden of CVD seems to differ greatly between men and women with T2D, and the excess cardiovascular (CV) risk observed in non-diabetic men compared to women is markedly reduced in the context of diabetes [10]. Moreover, prospective studies and meta-analyses suggest that there is a higher relative risk of CVD in T2D female patients [10,11,12,13]. Additionally, female patients show a higher risk of chronic kidney disease and end-stage renal disease compared to diabetic males [14,15,16,17], as well as a greater risk of cognitive impairment and cancer [18,19].

It is still unknown how the female gender could be responsible for the higher risk of both macro- and microvascular T2D complications. Among others, the higher body mass index (BMI) detected at the time of T2D diagnosis and the more frequent atypical presentations of coronary heart disease (CHD) in women can be taken into account [20,21,22]. Furthermore, different treatment strategies and drug metabolism are also contributors [23]. Additionally, a worse control of glycemia, lipid levels, and blood pressure has been reported in women compared to men, despite equal or even increased intensity of treatments [22,24,25,26,27].

Concerning disease management, women are more likely to achieve glycemic targets later than men after T2D diagnosis [28,29]. Both differences in drug efficacy and side effects are called into question for these outcomes. However, current studies about treatment outcomes between men and women still lack good female representation and are therefore unreliable [30,31].

Despite biological factors and different drug efficacy, medication adherence can be implicated in poor outcomes in females. Available records highlight that women have lower access to healthcare facilities due to social, cultural, and psychological issues [32,33]. A recent study has observed that Italian women have more difficulties in accessing diabetes care units compared to men [34]. Additionally, a lower number of T2D female patients are treated with antihyperglycemic agents in any age group compared to men [35]. When treated, women are also less frequently prescribed hypoglycemic agents with demonstrated cardio-renal protection [35]. All these data reveal significant sex disparities in T2D management, which may be causes of disease progression. Indeed, low medication adherence can have a powerful impact on morbidity and mortality in the management of chronic diseases [36,37,38,39,40,41].

Several observational studies, mostly retrospective, indicate the main determinants of non-adherence to antidiabetic treatment. On average, medication adherence was mainly related to clinical, sociodemographic, and system-level factors.

A retrospective study enrolling patients treated with oral antidiabetic agents reported an average percentage of medication adherence of 69%. The main predictors of low adherence were female sex, younger age, new drug prescription, low education level, and low social status [42]. A retrospective cohort analysis that evaluated medication adherence in T2D patients with newly prescribed oral antihyperglycemic agents showed quite similar results. This time, the predictors of low adherence were female sex, younger age, and non-white ethnicity. Additionally, adherence differed among the various types of drugs prescribed, being higher for metformin, while the non-adherence rate varied across other oral agents [43]. Data from real-world studies reported that female sex is an independent predictor of low medication adherence for both sulfonylureas and glucagon-like receptor agonists [44,45]. An even lower adherence is reported when patients are prescribed insulin. The percentage of adherence was 43% in patients newly prescribed basal insulin therapy, with the younger female patients showing the highest non-adherence rate [46,47].

Different studies have attempted to pinpoint adherence determinants of long-term oral antihyperglycemic therapy [48]. Indeed, adherence seems to be above 50% after three years from prescription and is higher in male patients (average age range of 50–60 years) and in therapy schemes involving more than three medications [48]. In addition to that, a longer disease duration (more than five years) has been reported as a predictor of good adherence, according to a recent large retrospective study [49].

A large analysis of medical claims involving patients treated for diabetes and CVD showed that women had lower medication adherence, were treated with more drugs, and were less likely to obtain guidelines-based prescriptions [50]. Thus, an observational Italian study reported that the percentage of patients with T2D over 65 years was higher in women compared with men (26.1% vs. 21.5%), highlighting that female T2D patients might have a higher clinical complexity due to their older age at the time of diagnosis [35]. The coexistence of multiple chronic diseases also seems to decrease medication adherence. In a large retrospective analysis, the coexistence of hypertension besides diabetes lowered the level of adherence compared to patients who only suffered from diabetes [51]. Similarly, the coexistence of diabetic complications appears to be another contributor to low adherence [52].

A meta-analysis of 22 studies revealed a gender gap in medication adherence to antihyperglycemic therapy. Depression, younger age, and female sex predicted low adherence [53]. Psychological disorders are common in T2D patients, with almost 30% experiencing depression [54,55]. Major depressive disorder rates are higher in diabetic patients, especially females, leading to significant consequences on metabolic control [56]. Diabetes distress affects patients’ self-management and clinical outcomes more than depression. [57,58,59]. This disorder indicates a whole range of feelings concerning comorbidities, complications, self-care, a sense of guilt, worries about hypoglycemia, or medical prescriptions. A recent study showed that healthcare professionals could help motivate patients, with women being more motivated than men when physicians used empathic communication [60]. In a randomized controlled trial, using informatics tools or educational printed items improved satisfaction and medication education [61,62]. Physicians’ effective communication is essential in helping patients and improving adherence, especially for patients with lower education or social background. Women showed more social barriers, leading to lower self-care adherence 63]. Conversely, perceived support was consistently related to better self-efficacy in women but not in men, even though men reported higher levels of support [63].

Another contributor to clinical outcomes in T2D patients is socioeconomic status. A recent systematic review and meta-analysis evaluated several studies showing that employment can lead to non-adherence to T2D treatment [64]. Thus, gender disparities may have an economic effect in terms of medication adherence and costs. In a large US study, a strong association was found between female sex and low medication adherence regarding costs: women were indeed more inclined than men to turn down medical prescriptions or delay medication replacements [65].

Over the last decades, the so-called “urban diabetes” has become a growing concern in wealthier areas of the globe [66]. Research should focus on better understanding how social background and gender can affect medication adherence.

In conclusion, reaching glycemic goals and controlling cardiovascular risk factors are well-known keys to diabetes care. Recently, the main clinical improvements in diabetes have resulted from the use of updated evidence-based standards of care and therapeutic algorithms, which are effective in reducing both mortality and costs. However, emerging barriers affecting diabetic clinical goals and contributing to a higher risk of diabetic complications and mortality need to be addressed. Among these, low medication adherence is recognized as one of the major determinants of poorer outcomes in diabetes management. Evidence and data from new studies have identified female gender as an independent predictor of low adherence to antidiabetic agents, as indicated by higher rates of worse clinical outcomes among female patients. Although the causes of this gender disparity are not completely understood to date, it is likely that a complex interplay of biological, clinical, sociodemographic, and psychological factors is involved. Strategies to improve medication adherence in T2D should consider these factors and adopt a personalized approach, taking into account the specific needs and challenges faced by women with diabetes. Empowering patients, providing effective communication, addressing psychological well-being, and addressing social determinants of health are crucial components in optimizing medication adherence and improving outcomes in T2D management (Table 1).

### 2.2. Hypercholesterolaemia

Hypercholesterolemia is a major risk factor for cardiovascular disease (CVD). Its prevalence is constantly rising worldwide, including in high-middle- and low-income countries [67]. High levels of low-density lipoprotein cholesterol (LDL-c) are estimated to be responsible for 3.78 million CV deaths and 0.61 million cerebrovascular deaths [68]. Notably, among all other CV risk factors, lipid alterations account for the majority of attributable risk for a first myocardial infarction in 49.5% of men and 47.1% of women, highlighting the predominant association between dyslipidemia and this disease [69]. Menopause and older age lead to a reduction in sex differences in lipid levels between men and women [70,71]. Specifically, only premenopausal women show a better lipid profile characterized by lower levels of LDL-c and higher levels of high-density lipoprotein cholesterol (HDL-c) compared to men [72].

Strong evidence from epidemiological studies and randomized controlled trials (RCTs) supports a logarithmic relationship between LDL-c variations and CVD risk [73,74,75]. It is well-established that there is a causal relationship between LDL-c and CVD, and LDL-c reduction therapy effectively reduces CVD risk [76]. Statins are the first-line pharmacological therapy for dyslipidemia [77]. Numerous meta-analyses have shown that statin use, both in primary and secondary prevention, is associated with a significant reduction in CV morbidity and mortality [73,78,79]. Notably, a large meta-analysis comparing statin therapy versus control and less intensive statin therapy found that each 1 mmol/L reduction in LDL-c achieved by statin therapy was associated with a 23% decline in the incidence of acute myocardial infarction (AMI), 20% reduction in CV death, 17% reduction in stroke, and 10% reduction in overall mortality over a period of 5 years [73].

Previous studies have debated the effectiveness of statins between men and women, particularly in the case of primary prevention [80,81]. This concern arose due to the relatively low percentage of women included in clinical trials investigating the CV efficacy of statins [82]. Typically, women tend to develop coronary artery disease 10 years later than men, which may partly explain their under-representation in clinical trials that primarily enrolled elderly patients [77]. As a result, the efficacy of statins in women has been questioned due to the limited number of gender-specific analyses [83]. The Cholesterol Treatment Trialists Collaboration analyzed 22 trials (174,149 participants, 27% women) to evaluate the effects of statin therapy on cardiovascular outcomes in both primary and secondary prevention for both men and women. After adjusting for confounding factors, the statistical analysis showed a similar reduction in major vascular events for both men and women, even in those with a predicted 5-year risk lower than 10%, suggesting equal efficacy of statins in both sexes [79].

Despite the demonstrated benefits of statin therapy, adherence to its prescription is not always optimal. The highest rate of discontinuation occurs soon after prescription and treatment adherence is estimated to be 50% at six months and 25% after one year [84]. Similarly, long-term adherence is not fully satisfactory, with discontinuation rates of 33% in primary prevention and 18% in secondary prevention observed in clinical trials after 5 years of treatment [85,86]. Suboptimal adherence to statin therapy has a significant impact on the incidence of CV events and mortality. Non-adherent patients have shown an increased risk of 1.22 to 5.26 for CV events and 1.25 to 2.54 for mortality in most observational studies [87]. Moreover, non-adherence is associated with a twofold higher risk of CV events and fourfold increased rates of stroke and death [88]. Various predictor factors have been identified, including socio-demographic factors (gender, age, ethnicity, income, education, costs), therapy-related factors (adverse events, statin type, and intensity, polypharmacotherapy), lifestyle factors (alcohol abuse), and patient perception (unawareness of the beneficial effects of treatment, medical distrust) [89] (Table 1).

Several observational studies and meta-analyses have attributed gender as a key factor influencing adherence to statin therapy, with evidence of poorer adherence among women compared to men. A recent Italian cohort study enrolled patients initiating statin therapy. After one year, the discontinuation rate was high in both sexes. Specifically, only 19% of women and 27% of men had a proportion of days covered (PDC, the ratio between the number of days when the medication is taken and the total number of days during the follow-up) higher than 80% (indicating optimal adherence) at one year. The gender difference was partly attenuated by age, as the male group had a higher mean PDC in all age groups up to 90 years. However, a higher percentage of male subjects with optimal adherence was observed only until 70 years, after which the proportion was higher in women [90].

A large meta-analysis evaluating 53 studies (including cohort studies, cross-sectional studies, and a few RCTs) found a higher percentage of non-adherent patients to statin therapy among women (53% of women and 50% of men). Female gender increased the risk of non-adherence by 10%. This excess risk was confirmed by studies that included multivariable models adjusting for other variables such as socioeconomic status, ethnicity, and indication for treatment (primary or secondary prevention). Additionally, non-white ethnicity was 53% more likely to be non-adherent compared to white ethnicity [91]. Another meta-analysis of 22 cohort studies reported that women were 7% more likely to be non-adherent than men. Furthermore, younger patients (under 50 years old) and older patients (over 70 years) were less adherent to therapy compared to those aged 50–65 years, indicating a U-shaped association between age and adherence. Other factors, such as higher income, secondary prevention, and comorbidities such as diabetes and hypertension, were associated with higher adherence [92]. A more recent meta-analysis, including 19 studies enrolling only primary prevention patients (two RCTs and mainly cohort and cross-sectional studies), confirmed higher adherence to statin therapy in men. In addition, obesity was associated with non-adherence only in women. Furthermore, a sex-dependent correlation between adherence and education was reported. Higher education was associated with higher adherence only in studies enrolling more than 50% men. Conversely, higher education was a predictor of low adherence in studies enrolling more than 50% women. This could be related to the different awareness of the risk of developing a CV event, mainly due to the widespread assumption that women have a lower CV risk compared to men. Diabetes and hypertension, higher income, previous smoking habits, and white ethnicity were predictors of good adherence. On the other hand, depression, alcohol abuse, and high-dose statins were correlated with non-adherence [93]. A large meta-analysis including only patients older than 65 years found different predictors of non-adherence, such as female gender, non-white ethnicity, current smoking habits, copayment, newly prescribed statins, primary prevention, depression, lower income, and polypharmacotherapy. In contrast, diabetes was associated with better adherence [94].

Many studies have found that statin-related side effects are a common cause of poor adherence [93,95,96]. Muscle symptoms are the most frequent adverse effects of statins. In RCTs, the occurrence of side effects, including statin-associated muscle symptoms (SAMS), was similar between the statin and placebo groups [97,98], and its prevalence is estimated to be around 7–29% in real-life settings [99,100,101]. Female sex is a known risk factor for SAMS, which significantly contributes to statin discontinuation [102] and might explain the association between female gender and poor adherence. Other factors that can worsen adherence include unawareness of the beneficial effects of statins, lack of knowledge about their mechanism of action, medical distrust, and lack of patient-physician communication [95,96].

Different strategies are useful for improving statin adherence, including better patient awareness, medical support, and doctor–patient relationship. Lastly, compelling evidence links female gender to poor adherence to lipid-lowering treatments. Variables such as socio-demographic factors, treatment-related factors, patient behavior, and perception-related factors are key contributors to medication adherence. Further research is needed to elucidate the correlation between these factors and their role in medication adherence (Table 1).

### 2.3. Arterial Hypertension

Hypertension is one of the major modifiable risk factors for cardiovascular disease (CVD) and a leading cause of mortality globally. Its prevalence is continually rising worldwide, although low- and middle-income countries have a more pronounced increase compared to higher-income countries [103,104]. In 2015, the global prevalence of high blood pressure was 1.13 billion, and the age-standardized prevalence was estimated to be 24% in men and 20% in women. Even though blood pressure (BP) is higher in younger male patients, this trend is inverted after 60 years of age, as the average increase in BP is greater in women [105]. Higher-income countries are associated with the lowest overall rate of women affected by hypertension. On the other hand, sub-Saharan Africa is associated with the highest prevalence of women with high blood pressure, mostly related to different lifestyle habits (diet and physical activity) among various socio-cultural contexts [103,106].

Hypertension is independently and linearly associated with CV morbidity and mortality at all ages and among all ethnic groups [107,108,109]. In 2015, hypertension-related CVD, hemorrhagic stroke, and ischemic stroke made hypertension the leading cause of disability and premature death, affecting almost 10 million people worldwide [110]. Additionally, hypertension is an independent risk factor for chronic kidney disease and end-stage renal disease [111]. Strong evidence from clinical trials showed that appropriate control of hypertension reduced the burden of CVD. Pharmacological intervention, combined with lifestyle education, is frequently required in hypertensive patients and is associated with a significant reduction in CV risk and mortality.

A large meta-analysis of randomized controlled trials (RCTs) showed that a 10 mmHg reduction in systolic BP and a 5 mmHg reduction in diastolic BP were associated with a reduction of 20% in all major CV events, 10–15% in all-cause mortality, 35% in stroke, 20% in coronary events, and 40% in heart failure, independently of age, gender, CV risk score, baseline BP values, and comorbidities such as diabetes and chronic kidney failure [112].

Despite the evidence, hypertension control remains far from optimal worldwide, and awareness of the disease is still limited. Real-life data show that BP goals are reached in less than 20% of all treated patients, whereas 80% of patients reached BP goals in clinical trials [113]. Additionally, real-life data show different evidence on BP treatment and outcomes between sexes compared to clinical trials [114,115].

An analysis from the National Health and Nutrition Examination Survey Mortality (NHANES) 1999–2004, which included patients taking antihypertensive medication, highlighted important differences between genders in BP treatment and control. When adjusted for age, ethnicity, and comorbidities, women with high BP were more frequently treated but were less likely to achieve BP goals, especially systolic BP, particularly at older ages and in the presence of comorbidities such as CVD, stroke, and chronic kidney disease. Partial BP control, especially at older ages, might explain part of the worse CV outcomes that affect women [116]. Diabetic women were more likely to be prescribed diuretics and angiotensin receptor blockers (ACE-Is). Furthermore, women with chronic kidney disease were less frequently treated with angiotensin receptor blockers (ARBs) compared to men [117].

Low adherence, along with suboptimal medical prescription and physician inertia, plays a crucial role in poor disease control [118,119]. The discontinuation of antihypertensive drugs is estimated to be up to 50% after one year, and low adherence to treatment may be responsible for more than 50% of resistant hypertension [120,121]. Poor adherence has an evident impact on CV outcomes, hospital admissions, and healthcare costs [122,123]. Conversely, different studies have demonstrated that better adherence to antihypertensive therapy is associated with improved outcomes. A large Italian prospective study showed that adherent patients had a 37% reduced risk of CV and cerebrovascular events compared to patients interrupting treatment over a six-year period [124]. Furthermore, a population-based cohort study including numerous patients on primary prevention starting antihypertensive treatment showed that high adherence was associated with a 56% decreased risk of a first CV event [125].

Identifying the most relevant risk factors associated with non-adherence is crucial to improving BP control and reducing the global burden of hypertension. Various determinants influence adherence to antihypertensive therapy, including socio-demographic factors (sex, age, ethnicity, income, and education), drug-related factors (acute or chronic adverse effects), clinical factors (presence of comorbidities leading to polypharmacotherapy and depressive disorders), patient’s disease and treatment awareness and knowledge, and factors related to the patient-physician relationship [126]. Detecting non-adherence is complicated due to the complex interplay among different contributors.

The scientific community has shown a growing interest in investigating the complex link between sex/gender and medication adherence, including hypertension therapy. Numerous observational studies, mainly based on pharmacy claims, have investigated hundreds of thousands of patients from different geographic areas to identify any correlation between sex differences and antihypertensive treatment compliance. However, the emerged data are inconclusive, showing opposite findings.

A large Italian population-based study that enrolled new users of antihypertensive drugs (50% women) showed that 30% of patients reported at least one episode of therapy interruption during a one-year follow-up. Males were associated with better adherence (53% vs. 42%), a 10% lower risk of discontinuation, and higher persistence, independently of age and type of medication. However, no difference emerged when patients with worse comorbidity status and taking drug combinations were compared [127]. Another Italian large cohort study, enrolling newly prescribed antihypertensive medication patients, demonstrated a lower rate of discontinuation in men, older patients, and patients on glucose-lowering medication with CVD or renal diseases. Conversely, depressive disorders and dementia were associated with a higher risk of discontinuation. Diuretic therapy was linked to the highest risk of interruption among drug classes [128].

A large Dutch population-based study found comparable results, with female sex being associated with a lower rate of adherence to antihypertensive therapy one year after its prescription [129]. A recent study collected urine samples from 174 patients (48% females) with poor BP control to evaluate medication adherence, despite the use of three or more BP-lowering medications. The overall non-adherence rate was 40%, and women had a three times higher probability of being non-adherent compared to men, after adjusting for confounders. Furthermore, a positive independent association between the number of medications and non-adherence was observed [130].

On the contrary, other observational studies showed lower adherence in men. A retrospective study observed factors such as male sex, dementia, history of stroke, and polypharmacotherapy to be associated with lower adherence [131]. Similarly, Friedman et al. studied drug adherence in a large sample of Canadian elderly patients initiating BP-lowering treatment. Female sex, absence of comorbidities, and high income were associated with higher compliance with treatment. Among drug classes, ACE-Is had the greatest rates of compliance, while beta-blockers had the worst [132]. Additionally, in a large US population-based cohort study of individuals older than 65 years who were newly prescribed antihypertensive medication the overall rate of low-intermediate adherence, measured as PDC, was around 40%. Factors such as female sex, non-Hispanic white ethnicity, use of more than one antihypertensive drug, and the presence of diabetes or dyslipidemia were associated with higher adherence [125]. A Swedish observational cohort study enrolled patients who received antihypertensive therapy for the first time. The data showed a low rate of treatment continuation both at one-year and two-year evaluations (57% and 43%, respectively). Risk factors for discontinuation included male sex, younger age, lower systolic BP at prescription, and lower income, with no difference observed between drug classes [133].

A recent meta-analysis collected data from 82 studies to evaluate adherence to BP-lowering medication using self-report or pharmacy refill prescription-based methods. After adjusting for confounding factors, no relation between sex and medication adherence was observed. These results were consistent across different geographic areas and adherence assessment methods. A subgroup analysis demonstrated higher adherence in men only in older age groups (>65 years) and studies adopting self-report methods for adherence assessment [134].

In conclusion, the role of sex as a determinant of medication adherence to antihypertensive treatment is still not fully established. The controversial data might be, at least partially, related to different methodological biases, such as the heterogeneity of methods selected for assessing adherence, differences in characteristics and cultures of the populations included, and discrepancies in the inclusion and conclusion criteria. Therefore, further research is still needed to clarify this issue (Table 1).

### 2.4. Cardiovascular Diseases

CVD is the leading cause of mortality worldwide, responsible for 17.9 million deaths each year [135]. Currently, more than three-quarters of these deaths are related to coronary heart disease (CHD) and stroke [135]. Traditionally, CVD has been considered more prevalent among men due to their higher incidence of CV events and mortality [136]. However, female cardiovascular risk seems to be delayed by approximately 10 years, and morbidity and mortality differences between sexes tend to diminish in older age, particularly for stroke [136]. Interestingly, more women than men die from CVD, largely due to their longer lifespan [77]. Conventional CVD risk factors have varying impacts on men and women, contributing to the sex and gender disparities in CV outcomes. For instance, women who smoke have a 25% higher risk of developing CHD compared to men who smoke [137]. Moreover, compelling evidence shows that diabetes has a greater impact on CV morbidity and mortality in women [138,139]. Female patients are reported to be less likely to achieve blood pressure (BP) targets and are more frequently undertreated than men. However, despite these differences, no clear gender disparities regarding the risk of adverse outcomes related to hypertension have been observed [116,117]. In addition to traditional risk factors, sex-related issues such as gestational diabetes and gestational hypertension play a critical role in increasing CV risk in women [140,141]. Furthermore, women have been noted to have poorer disease awareness, less social support, and a higher prevalence of depressive disorders. All these factors are believed to limit women’s access to care and widen sex inequalities [142]. Moreover, low socioeconomic status poses a greater additional CV risk in women compared to men [143].

Female CHD exhibits distinct pathophysiological features. Acute ischemia in women is commonly secondary to non-occlusive coronary lesions caused by microvascular damage [144], and acute myocardial infarction (AMI) typically manifests without ST elevation. Women also present with different clinical manifestations, including atypical symptoms such as weakness, dyspepsia, epigastralgia, dyspnea, and shoulder or back pain, which may contribute to delayed diagnosis and intervention [145]. Furthermore, in most studies, female patients have a higher risk of bleeding and vascular complications after percutaneous coronary intervention (PCI) [146,147]. Women also have a higher prevalence of atypical stroke symptoms, such as loss of consciousness, urinary incontinence, and swallowing difficulties [148]. Clear sex disparities have not emerged in studies focusing on acute treatment outcomes after stroke [149]. Randomized controlled trials (RCTs) evaluating evidence-based medications for CVD show equal efficacy in both sexes. However, the relatively low number of female participants enrolled in these studies limits the interpretation of their results [150,151,152].

It is important to consider that screening and management of CVD present sex disparities. In particular, women are less likely to be assessed for CV risk in primary care. They are also less frequently prescribed evidence-based medications recommended by current guidelines, such as beta-blockers, ACE inhibitors (ACE-Is) or angiotensin receptor blockers (ARBs), statins, and antiplatelet agents, for secondary prevention [153,154,155,156]. Furthermore, female patients less frequently achieve BP and lipid goals one year after AMI and experience more hospital readmissions than men [157]. Inequalities in healthcare may explain worse outcomes in women.

Optimal adherence is necessary to maximize the efficacy of evidence-based medications and to avoid poor CV outcomes. However, evidence shows that adherence to CV medication is far from optimal, leading to increased morbidity [158], mortality [159], and healthcare costs [158]. A meta-analysis of 20 studies, including 376,162 patients (51% female) in both primary and secondary prevention, evaluated adherence to seven drug classes (aspirin, ACE-Is, ARBs, beta-blockers, calcium channel blockers, thiazide diuretics, and statins). The data revealed an overall adherence rate of 57%, which did not exceed 50% and 66% for primary and secondary prevention, respectively [160]. Another meta-analysis examining 44 studies and 197,819 patients (23% on secondary prevention) investigated the effect of adherence to different drug classes (statins, antihypertensives, antiplatelet agents, antihyperglycemic medications, and other vascular agents) on CV events and all-cause mortality. Good adherence to medication was observed in only 60% of patients. Importantly, optimal adherence was associated with a 20% reduction in CVD and a 35% reduction in all-cause mortality [161].

Currently, limited data are available on sex inequalities in adherence to evidence-based medication regimens prescribed after AMI and stroke events. The few available studies demonstrate poorer adherence in women compared to men. In a recent retrospective study evaluating adherence to chronic pharmacological therapy (antiplatelet agents, statins, beta-blockers, ACE-Is, or ARBs) six months after discharge for a first AMI in 25,779 patients, overall adherence rates were 78% for statins, 59% for antiplatelet agents, 63% for ACE-Is/ARBs, and 50% for beta-blockers. However, full adherence was observed in only a quarter of patients, and women were 25% less likely to be adherent compared to men to evidence-based combined regimens post-AMI. Comorbidities and older age were predictive factors for low adherence [162]. Another Italian population-based cohort study evaluated adherence to antiplatelet agents, ACE-Is/ARBs, beta-blockers, and statins one year after AMI. The overall adherence rates were 90.5% for antiplatelet agents, 60% for beta-blockers, 78.1% for ACE-Is/ARBs, and 77.8% for statins [163].

A meta-analysis examining 44 studies (23% of patients with known CVD) evaluated the effect of various drug classes (statins, antihypertensives, antiplatelet agents, antihyperglycemic agents, and other vascular agents) on CV events and all-cause mortality. Only 60% of cases exhibited good adherence to evidence-based medication regimens. Importantly, optimal adherence was associated with a 20% reduction in CVD and a 35% reduction in all-cause mortality [161]. Currently, limited data are available on sex inequalities in adherence to evidence-based prescriptions after AMI or stroke. In general, most studies show poorer adherence in women compared to men. A recent Italian population-based retrospective study analyzed adherence to chronic medications (antiplatelet agents, statins, beta-blockers, ACE-Is, or ARBs) six months after discharge for a first AMI. The comprehensive adherence rates were 78% for statins, 69% for antiplatelet agents, 63% for ACE-Is/ARBs, and 50% for beta-blockers. However, only a quarter of patients were consistent with evidence-based combined regimens post-AMI, and female sex was associated with a 25% reduction in adherence compared to males, after adjusting for confounders. Additionally, factors such as older age and the presence of other comorbidities predicted lower adherence [162].

Authors from another Italian population-based cohort study investigated adherence to evidence-based pharmacological therapy, including antiplatelet agents, ACE-Is/ARBs, beta-blockers, and statins, one year after AMI. At the time of discharge, women were older and showed a worse comorbidity status compared to men. The overall adherence rates were 90.5% for antiplatelet medication, 60% for beta-blockers, 78.1% for ACE-Is/ARBs, and 77.8% for statins. After adjusting for confounders, women were 16% less likely to be adherent than men [163]. These findings were observed for both single drug classes and combined therapy. Older age was again a significant predictor of lower adherence [163]. Adherence to secondary prevention medications (antiplatelet agents, statins, beta-blockers, ACE-Is, or ARBs) and attendance of cardiac rehabilitation were recently examined six months and one year after discharge for acute coronary syndrome. After adjusting for confounders, women were more likely to be non-adherent to cardiac rehabilitation programs and showed a 35% increased risk of developing another major CV event after six months. After one year, women were less likely to be consistent with secondary prevention medications compared to men [164].

In a large retrospective cohort study, adherence to beta-blockers, ACE-Is/ARBs, and statins was investigated in patients after AMI. The overall adherence estimates one year after discharge were 66% for beta-blockers, 63% for ACE-Is/ARBs, and 66% for statins. Moreover, black women, and to a lesser extent, white women, had lower adherence to ACE-Is/ARBs, beta-blockers, and statins compared to white men one year after evaluation [165]. This trend was confirmed in a more recent retrospective cohort study of 52,672 patients, which found greater adherence to evidence-based drug prescriptions (antiplatelet agents/anticoagulants, beta-blockers, ACE-Is/ARBs) in male patients one year after AMI [166]. Along these lines, female gender and older age were significant predictors of non-adherence to secondary prevention therapies in previous studies [167,168].

On the other hand, sex differences in medication adherence after ischemic stroke are still insufficiently studied. A cohort study of patients older than 65 years evaluated adherence to antiplatelet therapy three years after a first ischemic stroke. The data showed that more than one-quarter of patients were not adherent, and women were 25% less likely to be persistent with therapy compared to men. Interestingly, in this case, older age (≥75 years) and other comorbidities such as diabetes were associated with better adherence to therapy [169].

The reasons for these sex differences in medication adherence after acute coronary syndrome and stroke are still largely unknown. Various factors, including sociodemographic, disease-related, treatment-related, and others, are believed to play a crucial role. Currently, there is growing interest in identifying potentially modifiable contributors, such as patients’ awareness, beliefs, and perceptions toward treatment, social support, and mood disorders (Table 1).

It should be highlighted that CVD is a multifaceted condition, ensuing from a rather intricate interplay of major and minor risk factors. Noteworthy, risk factors often coexist, being differently modulated by sex and gender. In this context, the relative impact of a single risk factor on CVD outcomes is rather complex to assess, as well as the effect of gender on multiple risk factors’ management in clinical practice. The coexistence of multiple chronic diseases seems to decrease medication adherence. Specifically, there are hints that female sex is associated with less adherence to medication schemes that target multiple risk factors. However, most studies have focused on adherence to medications after AMI (antihypertensive therapy, statins, antiplatelet agents). As regards the coexistence of diabetes and CVD, women treated for diabetes and CVD seem to have lower medication adherence compared to men [50], although further studies are needed to confirm this trend.

### 2.5. Heart Failure

Heart Failure (HF) is a widespread condition affecting 25 million people worldwide [170]. During the last decades, a slight reduction in age-standardized incidence has been observed. However, its overall prevalence has progressively increased, conceivably due to population aging and better survival rates after diagnosis [171]. Moreover, there has been a linear parallel increase in the prevalence of associated comorbidities, such as diabetes, hypertension, and CVD. These data suggest that the clinical presentation of patients with HF is becoming more complex, negatively affecting prognosis and mortality and further imposing a heavy burden on health services [171].

Women represent nearly half of the patients with HF [172]. Notably, a sex dimorphism in the clinical presentation of HF has been extensively described. In fact, women tend to be older than men at the time of diagnosis and are often associated with a poorer quality of life, a more complicated clinical phenotype, and severe and atypical symptoms [173]. Specifically, women have a two-fold increased risk of being affected by HF with preserved ejection fraction (HFpEF). On the other hand, men are more prone to suffer from HF with reduced ejection fraction (HFrEF). These findings suggest different etiologies and pathophysiological patterns between the sexes [174]. Particularly, hypertension is a common cause of HF in female patients, commonly leading to concentric cardiac hypertrophy, diastolic dysfunction, and HFpEF. In contrast, HF in men is more frequently associated with an ischemic etiology, which implies eccentric cardiac hypertrophy, dilatation, and reduced left ventricular EF [150].

Overall, mortality tends to be higher in males than in women, as a result of lower EF and a more frequent coexistence of CHD in men [175,176]. ACE-Is or an ARB and eventually added beta-blockers are part of the current evidence-based therapy for HF. A mineralocorticoid receptor antagonist (MRA) is added in patients with HFrEF with still uncontrolled symptoms. Diuretics are recommended in the presence of signs and symptoms of congestion. Sacubitril/valsartan is used as a replacement for ACE-I in patients with HFrEF with persistent symptoms despite optimal treatment with an ACE-I, beta-blocker, and MRA. Ivabradine is a treatment option in patients with left ventricular EF ≤ 35%, with sinus rhythm, and a heart rate ≥ 70 bpm despite treatment with a beta-blocker, ACE-I (or ARB), and MRA, or in patients who are unable to tolerate or have contraindications for beta-blocker treatment. Digoxin may be considered in symptomatic patients in sinus rhythm, despite treatment with an ACE-I (or ARB), beta-blocker, and MRA [177].

Recently, the anti-hyperglycemic class of Sodium-glucose co-transporter-2 (SGLT2) inhibitors has been shown to reduce the risk of hospitalization for HF in diabetic patients and also in patients without diabetes [178,179]. Current available data show sex differences in drug safety and efficacy [150]. In particular, considering relevant sex disparities in pharmacokinetics and pharmacodynamics profiles, women develop drug side effects more frequently [180]. For instance, the development of cough and angioedema ACE-Is seems to be higher in women than in men [181]. Additionally, diuretic therapy more frequently leads to electrolyte disorders in women compared to men [180]. Taking into account drug efficacy, it is noteworthy that women’s proportion in RCTs, as well as in preliminary studies for drug development and dose assessing, is scarce, generally not exceeding 20–30% [150,151,152]. Furthermore, sex-stratified analyses are either not performed or underpowered to detect significant differences.

Patients with HF have a poor prognosis and high mortality. In fact, the 1-year all-cause mortality rate is estimated to be around 6.4%, while the combination of mortality or hospitalization within 1 year is 14.5% [182]. The aforementioned evidence-based HF pharmacological treatment has been widely demonstrated to reduce adverse outcomes in large RCTs [183]. Additionally, adequate self-care measures, mostly changes in dietary habits, weight and fluid monitoring, and optimal medication adjustments, can lead to a significant improvement in prognosis if adopted [184].

Like other chronic disorders, adherence to HF drug prescriptions plays a crucial role in achieving treatment goals and reducing the burden of the disease. It has been observed that each 10% increase in the proportion of days covered (PDC) significantly reduces hospital admissions and all-cause mortality [185]. Accordingly, two large meta-analyses evaluated the efficacy of several types of intervention to improve medication adherence, such as training/education, reminder tools, technical measures, and medical support. The results showed a significant reduction in mortality by 2–11% and in hospitalization by 10–21% [186,187].

Evidence about sex/gender differences in adherence to evidence-based HF treatment is still limited. Only a few studies have investigated this issue, leading to conflicting results. A large cohort study involving patients with HF (47.9% women), newly prescribed with an evidence-based HF drug regimen, studied adherence to therapy. Notably, men were significantly less likely than women to be adherent one year after initiation [188]. Moreover, in a retrospective cohort study, women were more adherent to ACEIs/ARBs therapy after their first hospital admission for HF. In addition, a larger number of comorbidities was associated with a higher adherence to these drugs. Conversely, adherence to beta-blockers was not influenced by these factors [189]. Similarly, in a population-based study enrolling patients treated with conventional medications for HF (41% female), males were more likely to be non-adherent to ACE-Is/ARBs compared to women, but this relationship between sex and adherence was not observed for other drug classes [190]. Another large retrospective study reported lower adherence to HF treatment in male patients [191].

On the other hand, Granger et al. found opposite results by analyzing adherence in patients with HF enrolled in the Candesartan in Heart Failure Assessment of Mortality and Morbidity (CHARM) trial (n = 7599, 31.5% women). Particularly, women were less adherent compared to men after adjusting for confounders, and this difference was even more marked when considering women younger than 75 years. This trend remained significant both in the candesartan and in the placebo arm. Of note, women were prescribed a higher number of drugs even though fewer evidence-based medications were adopted, as beta-blockers were less prescribed, while the use of calcium blockers was more common among them compared to men [192]. Similarly, in a sample of 236 patients with HF (35.2% women), other authors reported higher adherence to ACE-Is in men compared to women six months after hospital discharge [193]. However, a recent retrospective study including 25,776 patients with HF (45% women) did not find any difference between men and women in adherence to medication [194].

In addition, adherence to self-care recommendations (weight monitoring, fluid and sodium restriction, and physical activity) has shown conflicting data about its association with sex. In a cross-sectional study, adherence to self-care recommendations was evaluated in a sample of 310 patients with HF (64.2% women). Men were significantly more adherent compared to women after adjustment for confounders. The absence of comorbidities and a high level of knowledge of the disease resulted in other predictors of good adherence [195]. Some authors reported similar results [196,197], while other authors observed no significant sex differences [198,199].

Overall, the evidence that examined the effect of sex/gender on adherence to HF therapy is still insufficient to draw firm conclusions. In consideration of the relevant impact of medication adherence on HF outcomes, further research is needed on this issue (Table 1).

## 3. Conclusions

It is, therefore, crucial to assess adherence levels in clinical settings using reliable and cost-effective tools and to identify risk factors for non-adherence through large-scale studies. This approach aims to achieve complete adherence to treatment and successful management of chronic diseases (Table 2).

Interest in understanding the impact of sex and gender on medication adherence and identifying modifiable barriers, including cognitive, mood-related, and psychosocial factors, has grown in recent decades. Emerging evidence reveals a sex dimorphism in medication adherence, which could partly explain higher rates of poor outcomes in women compared to men for certain chronic conditions. Studies and meta-analyses demonstrate that being female is an independent predictor of non-adherence to antidiabetic medications, lipid-lowering therapy, and evidence-based medication regimens after acute myocardial infarction (AMI). However, data regarding sex disparities in hypertension, heart failure (HF), and stroke is limited and conflicting due to the scarcity of available studies.

The underlying reasons for this dimorphism in medication adherence are largely unknown. Several factors, such as biological, treatment-related, psychosocial, socioeconomic, cognitive, and mood-related aspects, and their complex interplay, may contribute to sex disparities. Women are more likely to experience drug side effects. Feasible solutions to overcome this barrier include the adequate implementation of treatment plans and therapeutic interchange. Moreover, numerous studies have identified complex medication regimens as negative predictors of adherence. Therefore, utilizing combination pills and long-acting drugs, and avoiding complex drug regimens, could be a useful approach to address treatment-related issues. Notably, women appear to have lower awareness of their cardiovascular risk, harbor more negative perceptions and beliefs about diseases and treatment, receive less social support, and experience higher rates of depressive disorders. Additionally, the common role of women as caregivers within the family context might negatively impact their self-care (Table 2).

Despite limited studies investigating the role of these factors and lacking definitive conclusions, healthcare providers often overlook these variables. Routine assessment of these factors can effectively help overcome adherence barriers and improve outcomes. Factors such as patients’ unawareness, misperception of treatment benefits, and psychological barriers could significantly benefit from improvements in the doctor–patient relationship and enhanced medical support. Given these findings from observational studies, it is essential to conduct intervention studies with a gender-centered design and appropriate sample sizes. This approach will help identify effective solutions for promoting adherence in a clinical setting (Table 2). Increasing awareness and knowledge about sex and gender disparities can lead to more gender-customized interventions and tailored clinical approaches, thereby significantly improving outcomes and substantially reducing the burden of metabolic and cardiovascular diseases (Table 3).

## Figures and Tables

**Table 1:** Observations of non-adherence through all the conditions examined.

Type of Condition	Observations
Type 2 Diabetes	Women show low medication adherence to anti-hyperglycemic treatments. Depressive disorders and diabetes distress are significantly more common in female patients and seem to play a key role
	Women with diabetes might greatly benefit from more structured and supportive educational programs, possibly involving multidisciplinary teams, aimed at overcoming barriers to medication adherence
Dyslipidemia	Non-adherence is due to several factors (mainly socio-demographic and treatment-related) and appears to be more frequent in women
	New treatment strategies are needed to improve adherence (association therapy, therapeutic interchange, increased medical support)
Arterial Hypertension	Women are less likely to achieve Blood Pressure targets
	The contribution of sex as a determinant of medication adherence is still controversial
Cardiovascular Disease	Worse outcomes in cardiovascular diseases among women could be associated with disparities in health assistance, including risk assessment and evidence-based medication prescription
	Most studies are consistent with poorer adherence in women, but the reasons are largely unknown and involve a complex overlap between numerous factors
Heart Failure	Studies that examined the effect of sex/gender on adherence to heart failure therapy are still insufficient to draw firm conclusions
	In consideration of the relevant impact of medication adherence on heart failure outcomes, further research is needed on this issue

**Table 2:** Causes of reduced medication adherence and proposed strategies to improve adherence.

Causes of Non-Adherence	Suggested Strategies to Improve Adherence
Complexity of treatment, polypharmacy	Single pill administration
Patient’s misperception	Improve patient awareness and doctor–patient relationship
Lack of benefits in treatment or immediacy of beneficial effects	Increase availability of medical support
Poor relationship patient-doctor	
Psychological problems, cognitive Impairment	Role of caregivers
Documented side effects	Implementation of treatment plan
	Therapeutic interchange

**Table 3:** Studies that evaluated gender-related factors in medication adherence.

Type 2 Diabetes		
Authors	Year	Main Findings
Bird CE, et al. [32]	2007	Women have lower access to healthcare facilities due to social, cultural, and psychological issues
Fisher L, et al. [58]	2010	Diabetes distress affects patients’ self-management and clinical outcomes more than depression
Penno G, et al. [27]	2013	Women with type 2 diabetes have worse control of glycemia, lipid levels, and blood pressure despite equal or increased treatment intensity
Malmenas M, et al. [45]	2013	Female sex is an independent predictor of low medication adherence for glucagon-like receptor agonists
Manteuffel M, et al. [50]	2014	Women have lower medication adherence, are treated with more drugs, and are less likely to obtain guidelines-based prescriptions
Kirkman MS, et al. [42]	2015	The main predictors of low adherence are female sex, younger age, new drug prescription, low education level, and low social status
Mansyur CL, et al. [63]	2015	Women show more social barriers and less support, leading to lower self-care adherence
Iglay K, et al. [44]	2016	Female sex is an independent predictor of low medication adherence for sulfonylureas
Brunton SA, et al. [41]	2017	Low adherence is associated with a higher hospitalization rate and a negative impact on costs
Hofer R, et al. [62]	2017	There is a strong relationship between improved satisfaction with medication knowledge and increased adherence
Kim YY, et al. [36]	2018	Low adherence to antihyperglycemic medications is associated with an increased risk of all-cause mortality and cardiovascular events
McGovern A, et al. [43]	2018	Adherence differs among various types of drugs prescribed, being higher for metformin, while non-adherence rate varies across other oral agents
Choi YJ, et al. [53]	2018	Younger age, female sex, and depression are predictors of low adherence
Bhaloo T, et al. [60]	2018	Women are more motivated than men when physicians use empathic communication
Bhuyan SS, et al. [65]	2018	Female sex is associated with low medication adherence due to cost-related factors
Horii T, et al. [48]	2019	Adherence is higher in male patients and in therapy schemes involving more than three medications
Xu N, et al. [49]	2020	Longer disease duration (more than five years) is a predictor of good adherence
Demoz GT, et al. [52]	2020	The coexistence of diabetic complications is a contributor to low adherence
Aronson BD, et al. [59]	2020	Diabetes distress and depressive disorders, more frequent in females, have a role in low medication adherence, suggesting an implication in sex disparities
Beernink JM, et al. [40]	2021	Medication adherence is important to control healthcare system costs arising from hospitalizations due to disease progression and complications
Jankowska-Polanska B, et al. [51]	2021	The coexistence of hypertension alongside diabetes lowers the level of adherence compared to patients who only suffer from diabetes
Hypercholesterolaemia		
Mann DM, et al. [92]	2010	Women were 7% more likely to be non-adherent than men
Lewey J, et al. [91]	2013	Female gender increased the risk of non-adherence by 10%
Stroes ES, et al. [102]	2015	Female sex is a known risk factor for SAMS, which significantly contributes to statin discontinuation
Ofori-Asenso R, et al. [94]	2018	Female gender was associated with lower adherence to statin therapy among older patients (>65 y.o.)
Hope HF, et al. [93]	2019	Male gender was associated with higher adherence to statin therapy for primary prevention
Ingersgaard MV, et al. [89]	2020	Gender is one of the main predictors of low adherence
Olmastroni E, et al. [67]	2020	Women showed lower adherence to statin therapy after initiation
Arterial hypertension		
Erkens JA, et al. [129]	2005	Female gender was associated with a lower rate of adherence to antihypertensive therapy one year after its prescription
Brown DW, et al. [107]	2007	Women with high blood pressure were more frequently treated but were less likely to achieve blood pressure goals, especially in systolic blood pressure, particularly at older ages and in presence of comorbidities such as CVD, stroke, and chronic kidney disease
Friedman O, et al. [132]	2010	Female sex, absence of comorbidities, and high income were associated with higher compliance with antihypertensive treatment among elderly patients
Mancia G, et al. [128]	2014	Males showed better adherence to blood pressure therapy and a 10% lower risk of discontinuation
Tajeu, et al. [131]	2016	Male sex was one of the risk factors of lower adherence to antihypertensive treatment
Qvarnstrom M, et al. [133]	2016	Male sex, younger age, lower systolic blood pressure at prescription, and lower income were related to lower adherence to antihypertensive treatment in newly prescribed patients
Burnier M [126]	2017	Gender is among determinants influencing adherence to antihypertensive therapy
Yang Q, et al. [125]	2017	Female sex, non-Hispanic white ethnicity, use of more than one antihypertensive drug, and the presence of diabetes or dyslipidemia were associated with higher adherence
Rea F, et al. [127]	2020	Women were associated with higher rates of antihypertensive therapy interruption after first prescription
Biffi A, et al. [134]	2020	No relation between sex and medication adherence was observed. A subgroup analysis showed higher adherence in men only in older age groups (>65 y)
Cardiovascular Diseases		
Kirchmayer U, et al. [163]	2012	The adherence rates were 90.5% for antiplatelet agents, 60% for beta-blockers, 78.1% for ACE-Is/ARBs, and 77.8% for statins; women were 16% less likely to be adherent than men
Lauffenburger JC, et al. [165]	2014	Black women, and to a lesser extent, white women, had lower adherence to ACE-Is/ARBs, beta-blockers, and statins compared to white men
Backholer K, et al. [143]	2017	Low socioeconomic status poses a greater additional cardiovascular risk in women compared to men
Goldstein JM, et al. [142]	2019	Women have poorer disease awareness, less social support, and a higher prevalence of depressive disorders, contributing to limited access to care and widening sex inequalities
Carcel C, et al. [149]	2020	Clear sex disparities have not emerged in studies focusing on acute treatment outcomes after stroke
Soldati S, et al. [162]	2021	Comorbidities and older age were predictive factors for low adherence
Hyun K, et al. [153]	2021	Women were less likely to be consistent with secondary prevention medications compared to men
Heart failure		
Roe CM, et al. [193]	2000	Men showed higher adherence to ACEIs six months after hospital discharge
Bagchi AD, et al. [191]	2007	Male patients were less adherent to HF treatment
Lamb DA, et al. [189]	2009	Women were more adherent to ACEIs/ARBs therapy after their first hospital admission for heart failure
Granger BB, et al. [192]	2009	Women were less adherent compared to men to HF treatment. This difference was more consistent considering women younger than 75 years
Dunlay SM, et al. [190]	2011	Males were more likely to be non-adherent to ACEIs/ARBs compared to women, but this relationship between sex and adherence was not observed for other drug classes
Kayibanda JF, et al. [188]	2018	Men were less likely than women to be adherent one year after initiation of evidence base HF drug regimen
Gurgoze MT, et al. [194]	2021	No Sex difference in adherence to HF medication was found
